# A novel model for predicting prognosis in pancreatic cancer patients: a retrospective study

**DOI:** 10.3389/fonc.2025.1622821

**Published:** 2025-12-26

**Authors:** Mengyi Jiang, Meixiang Zhou

**Affiliations:** Department of Medical Oncology, Shanghai Sixth People’s Hospital Affiliated to Shanghai Jiao Tong University School of Medicine, Shanghai, China

**Keywords:** inflammation, overall survival, pancreatic cancer, prognostic prediction, tumor marker

## Abstract

**Background:**

Pancreatic cancer is notoriously associated with a poor prognosis and limited survival. We aim to develop a simple and accessible model that can accurately predict the prognosis of pancreatic cancer patients.

**Methods:**

This study retrospectively analyzed the blood indicators and overall survival of 500 pancreatic cancer patients. The median value was used as the cutoff for univariate and multivariate analyses. To address the limitations of the median value, receiver operating characteristic analysis was performed, and the optimal cutoff value (the highest Youden index) was determined, followed by univariate and multivariate analyses. Prognostic LASSO coefficient screening was performed to establish a pancreatic cancer prognostic prediction model. Risk factor diagram, Kaplan-Meier curve and prognostic calibration curve were plotted to validate the efficacy of the model.

**Results:**

Multivariate regression analysis showed that neutrophils (hazard ratio (HR) = 1.416, 95% confidence interval (CI) = 1.037-1.932, *P* = 0.028), lymphocytes (HR = 0.625, 95% CI = 0.462-0.846, *P* = 0.002), Carcinoembryonic Antigen (CEA) (HR = 1.820, 95% CI = 1.315-2.518, *P* < 0.001), CA125 (HR = 1.392, 95% CI = 1.001-1.936, *P* = 0.049), TNM stage (I vs. III: HR = 3.052, 95% CI = 1.900-4.905, *P* < 0.001; I vs. IV: HR = 4.815, 95% CI = 2.504–9.258, *P* < 0.001) and Neutrophil-to-Lymphocyte Ratio (NLR) (HR = 1.748, 95% CI = 1.210–2.525, *P* = 0.003), Lymphocyte-to-Monocyte Ratio (LMR) (HR = 0.597, 95% CI = 0.430–0.829, *P* = 0.002), Neutrophil-to-Macrophage Ratio (NMR) (HR = 2.065, 95% CI = 1.331–3.206, *P* = 0.001), and Systemic Immune-Inflammation Index (SII) (HR = 1.751, 95% CI = 1.244–2.466, *P* = 0.001) were independent risk factors for OS. We have developed a new model incorporating gender, age, treatment, TNM stage, pathological grade, CEA, CA125, and NLR. The model demonstrates good predictive performance, with a C-index of 0.73.

## Introduction

Pancreatic ductal adenocarcinoma (PDAC) is one of the four leading causes of cancer related death and is predicted to become the second most common cause by 2030 (1). Despite great research progress in recent decades, the overall 5-year survival rate is still nearly 10% (2). Due to its anatomical location, PDAC is often diagnosed at advanced stages, making surgery impossible for the majority of patients (3).

Inflammation is described as hallmark of cancer and is implicated in tumor development and malignant progression. In PDAC, inflammatory cells, such as macrophages and neutrophils, release large amounts of reactive oxygen species (ROS) and reactive nitrogen species (RNS) (4). These molecules can directly damage DNA, proteins, and lipids. Persistent oxidative stress leads to DNA damage, and if the repair mechanisms fail, it may trigger mutations in oncogenes (such as KRAS), thereby promoting the development of PDAC (5). ROS can also activate pro-inflammatory and pro-cancer signaling pathways, such as NF-κB and STAT3, further driving cell proliferation and survival (6).Pancreatic cancer cells secrete chemokines (such as CCL2 and CXCL8), which attract inflammatory cells like macrophages and neutrophils into the tumor microenvironment. These inflammatory cells release additional cytokines and growth factors, further stimulating cancer cell proliferation, invasion, and angiogenesis. Chronic inflammation leads to fibrosis of pancreatic tissue, creating a hypoxic environment that promotes cancer cell adaptation to harsh conditions and enhances their invasiveness (7). Inflammation facilitates PDAC development through mechanisms such as oxidative stress, cell proliferation, inhibition of apoptosis, and immune suppression. Simultaneously, it interacts with the tumor microenvironment, driving cancer cell invasion, metastasis, and treatment resistance (8, 9).

Clinical studies have shown that the levels of neutrophils, macrophages, and lymphocytes are associated with the prognosis of PDAC patients (10, 11). The establishment of related models such as Neutrophil-to-Lymphocyte Ratio (NLR), Neutrophil-to-Macrophage Ratio (NMR), and Systemic Immune-Inflammation Index (SII) can assist in predicting the prognosis (12–14).

We collected pre-treatment blood indicators from 500 PDAC patients and found that neutrophils, lymphocytes, NLR, Lymphocyte-to-Monocyte Ratio (LMR), NMR, SII were statistically significant in relation to overall survival (OS). Multivariate analysis incorporating treatment, TNM stage, and other clinical characteristics demonstrated that CEA held higher prognostic predictive value than CA199. By calculating the LASSO coefficients to identify the optimal combination, we established a model that integrates inflammatory indicators and tumor markers. This model can more effectively predict the prognosis of PDAC patients based on blood indicators and clinical characteristics.

## Methods

### Patient population

Patients with PDAC hospitalized at the Sixth People’s Hospital Affiliated with Shanghai Jiao Tong University School of Medicine, from January 2014 to December 2022, were retrospectively screened. The inclusion criteria indicated below: (1) pathological diagnosis of pancreatic ductal adenocarcinoma; (2) no loss to follow-up; (3) relatively complete clinical data. The exclusion criteria were as follows: (1) pathological diagnosis of pancreatic neuroendocrine carcinoma or pancreatic acinar cell carcinoma; (2) patients with second or multiple primary cancers; (3) lack of necessary data (detailed clinicopathological data or follow-up data). A total of 500 patients who met our inclusion criteria, with clinicopathological data collected from medical records, were included in our study.

### Data collection

clinical variables of patients, including age, sex, follow-up time and status, clinical stage, pathological grade, and history of therapy (radical surgery or palliative chemotherapy), activated partial thromboplastin time (APTT), prothrombin time (PT), international normalized ratio (INR), thrombin time (TT), fibrinogen (Fg), fibrin degradation products (FDP), d-dimer (D-D), alanine aminotransferase (ALT), aspartate aminotransferase (AST), total bilirubin (TBiL), direct bilirubin (DBiL), albumin (ALB), globulin (GLB), creatinine (Cr), blood urea nitrogen (BUN), alpha-fetoprotein (AFP), CEA, quamous cell carcinoma antigen (SCC), cancer antigen 125 (CA125), cancer antigen 724 (CA724), cancer antigen 199 (CA199), cancer antigen 242 (CA242), white blood cell count (WBC), neutrophil (NEUT), lymphocyte (LYMPH), monocyte (MONO), red blood cell (RBC), hemoglobin (Hb), mean corpuscular volume (MCV), red cell distribution width (RDW), platelet count (PLT), were collected. Laboratory data were obtained from routine blood tests performed at the time of initial diagnosis. NLR, LMR, PLR, NMR and SII were calculated through the following formula: NLR = neutrophil/lymphocyte; LMR = lymphocyte/monocyte; PLR = platelet/lymphocyte; NMR = neutrophil/monocyte; SII = platelet × neutrophil/lymphocyte, respectively. Telephone follow-ups were conducted for the included patients to obtain their OS data. When *P* < 0.05, the variable is considered statistically significant for prognosis.

### Statistical analysis

Excel was utilized for data management, while R software was employed for analyzing the data. Categorical variables were summarized using absolute frequencies and percentages, whereas continuous variables were presented as medians with ranges. A risk regression model was applied to evaluate the relationship between blood indicators and prognosis. To circumvent the limitations inherent in median-based dichotomization, time-dependent receiver operating characteristic (ROC) analysis was performed, and the optimal cutoff value (the highest Youden index) was determined. Univariate and multivariate Cox regression analyses were conducted to identify variables that have a significant impact on prognosis, while hazard ratio (HR) and 95% confidence interval (CI) were calculated. The optimal prognostic model was developed by employing LASSO regression with three-fold cross-validation, using the Concordance Index (C-index) as the evaluation metric. A risk score plot was subsequently constructed based on this prognostic model. Kaplan-Meier curve and prognostic calibration curve were plotted to validate the efficacy of the model.

### Ethical considerations

Ethical permission was approved by Ethics Committee of Shanghai Sixth People’s Hospital. The study protocol was designed in accordance with Good Clinical Practice and conducted in compliance with the Declaration of Helsinki. All participants provided written informed consent prior to their inclusion in the study.

## Results

### Baseline characteristics of patients

**Figure 1:**
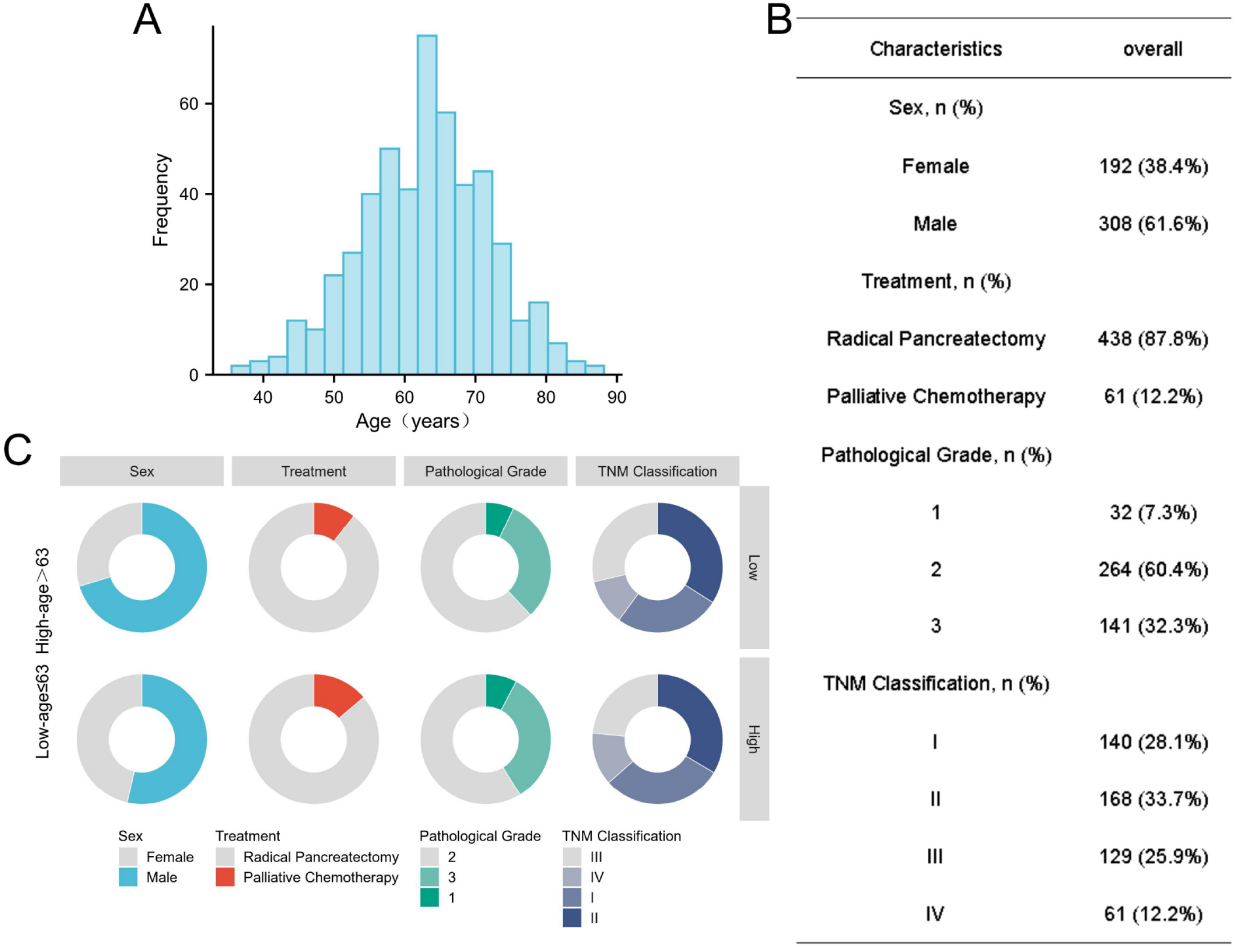
**(A)** Age distribution of 500 PDAC patients. **(B, C)** Clinicopathological characteristics of 500 PDAC patients.

This study included a total of 500 PDAC patients, with a median follow-up time of 12 months. The shortest OS was 1 month, and the longest OS was 39 months. By the last follow-up, 215 patients (43%) had died. Among these patients, there were 308 females and 192 males. A total of 438 patients underwent radical resection for pancreatic cancer, while 61 patients, who were not candidates for surgery, received palliative chemotherapy (**Figure 1**).

### Univariate cox regression analysis when the cutoff value was median

To establish a prognostic prediction model for pancreatic cancer, we analyzed the median values and ranges of collected blood indicators (**Table 1**). Using the median values as cutoff points, we performed univariate Cox regression model analysis to assess their association with prognosis. The following factors, which were showed in **Table 2**, were found to have a statistically significant association with prognosis: age, TNM stage, treatment, FDP, D-dimer, CEA, CA125, CA199, CA242, NEUT and LYMP (*P* < 0.05).

**Table 1:** The characteristics of the blood indicators.

Category	Mean (SD)	Median (range)
APTT (s)	29.13 (3.55)	28.3 (17-51.6)
PT (s)	11.94 (6.07)	11.5 (1.02-143.6)
INR	0.99 (0.1)	0.98 (0.82-2.07)
TT (s)	17.85 (1.37)	17.7 (14.3-28)
Fg (g/L)	3.19 (0.86)	3.0 (1.41-8.32)
FDP (mg/L)	2.9 (9.0)	1.4 (0-184)
D-D (mg/L)	0.74 (2.33)	0.35 (0.1-47.18)
ALT (IU/L)	107.57 (159.41)	28.0 (5.0-1148.0)
AST (IU/L)	78.63 (116.07)	25 (8.7-1098)
TBiL (umol/L)	69.16 (99.50)	17.0 (5.0-532.6)
DBiL (umol/L)	36.21 (58.83)	3.3 (0-281)
ALB (IU/L)	38.78 (5.83)	39 (0-72)
GLB (IU/L)	29.34 (5.49)	29.08 (0-50.82)
Cr (umol/L)	67.98 (17.43)	66.0 (1.14-199)
BUN (mmol/L)	6.73 (18.4)	4.9 (1.4-293)
AFP (ng/mL)	3.16 (2.04)	2.72 (0-23.87)
CEA (ng/mL)	7.21 (20.07)	3.46 (0.6-290)
SCC (ng/mL)	0.95 (1.53)	0.7 (0.2-22)
CA125 (U/mL)	33.31 (91.26)	16.3 (3.5-1769.3)
CA724 (U/mL)	5.65 (24.56)	1.84 (0.2-300)
CA199 (U/mL)	843.3 (2569.71)	163.8 (0.6-20460)
CA242 (U/mL)	54.79 (68.46)	19.1 (0.01-220)
WBC (× 10^9/L)	5.97 (2.22)	5.5 (2.23-19.92)
NEUT (× 10^9/L)	3.89 (1.98)	3.46 (0.09-18.01)
LYMPH (× 10^9/L)	1.49 (0.73)	1.39 (0.05-10.28)
MONO (× 10^9/L)	0.43 (0.18)	0.39 (0.06-1.56)
RBC (× 10^12/L)	4.22 (0.57)	4.24 (1.3-8.5)
Hb (g/L)	129.65 (17.87)	130.0 (4.02-206)
MCV (fl)	91.6 (6.49)	91.80 (3.62-104.9)
RDW (%)	13.72 (1.68)	13.3 (11.3-31.5)
PLT	192.7 (69.52)	187.0 (41-542)
AGE (years)	62.81 (9.04)	63 (37-87)
OS (months)	13.89 (8.99)	12 (1-39)

**Table 2:** Univariate Cox regression analysis of OS in PDAC patients.

Characteristics	HR	95%CI	P value
Age			
≤63	1	Reference	
>63	1.5057	1.151-1.97	0.00283 **
TNM			
I-II	1	Reference	
III-IV	2.4715	1.884-3.242	6.29e-11 ***
treatment			
palliative	1	Reference	
radical	0.3625	0.2628-0.5	6.22e-10 ***
FDP			
≤1.4mg/L	1	Reference	
>1.4mg/L	1.5106	1.129-2.021	0.0055 **
D-D			
≤0.35mg/L	1	Reference	
>0.35mg/L	1.5797	1.183-2.109	0.00193 **
CEA			
≤3.46ng/mL	1	Reference	
>3.46ng/mL	1.6438	1.247-2.168	0.000429 ***
CA125			
≤16.3 U/mL	1	Reference	
>16.3 U/mL	2.0596	1.532-2.769	1.74e-06 ***
CA199			
≤163.8 U/mL	1	Reference	
>163.8 U/mL	1.6122	1.225-2.121	0.000644 ***
CA242			
≤19.1U/mL	1	Reference	
>19.1U/mL	1.7865	1.313-2.43	0.000218 ***
NEUT			
≤3.46× 10^9/L	1	Reference	
>3.46× 10^9/L	1.3651	1.043- 1.787	0.0235 *
LYMPH			
≤1.39× 10^9/L	1	Reference	
>1.39× 10^9/L	0.7520	0.5742-0.985	0.0385 *
MONO			
≤0.39× 10^9/L	1	Reference	
>0.39× 10^9/L	1.3011	0.9921-1.706	0.057

* : P < 0.05; ** : P < 0.01; *** : P < 0.001.

### The optimal cutoff values calculated by Youden index method

**Figure 2:**
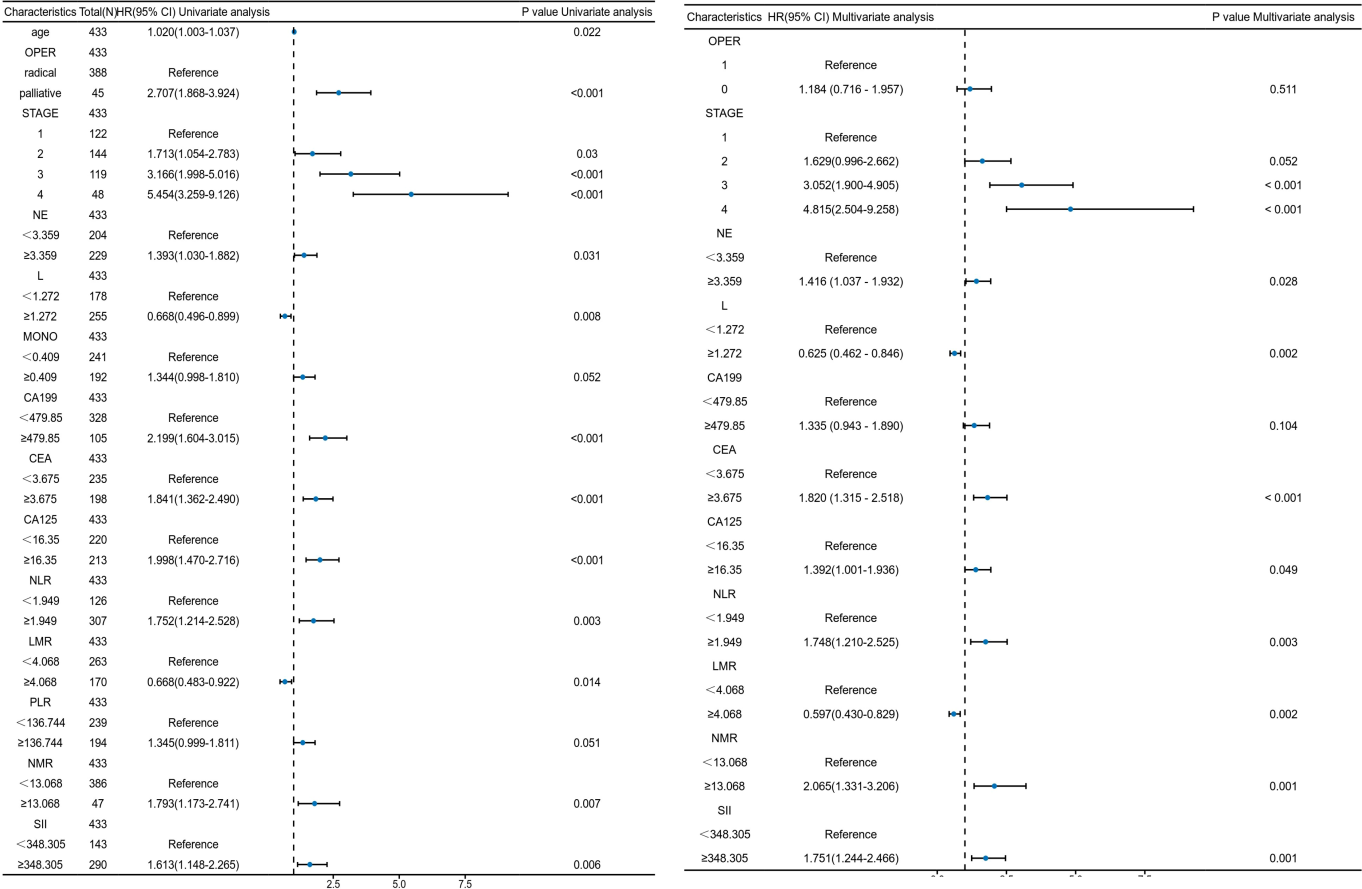
Forest plot of univariate and multivariate analyses for PDAC patients. (OPER: 1, Radical Pancreatectomy; 0, Palliative Chemotherapy).

To avoid the potential bias associated with using the median value as the cutoff, we additionally plot time-dependent ROC curves for the highest Youden index and identify the optimal cutoff values, which maximizes the difference between sensitivity and specificity to identify the most effective threshold for prognostic prediction (**Table 3**). Univariate Cox proportional hazards regression models were used to assess the association between patient characteristics and OS, and the results were visualized using a hazard ratio forest plot (**Figure 2**). The results showed that among the clinical characteristics, TNM stage and treatment had a significant impact on the prognosis of PDAC patients. Among the blood indicators, neutrophil, lymphocyte, CA199, CA125, CEA, NLR, LMR, NMR, and SII were statistically significant in relation to prognosis.

**Table 3:** The optimal cutoff values of blood indicators and the median survival time.

Factor	Cut-Off	mOS (Months) ≥Cut-Off	mOS (Months) <Cut-Off
Neutrophils (/nL)	3.359	11	13
Lymphocytes (/nL)	1.272	13	11
Monocytes (/nL)	0.409	11	12
Platelets (/nL)	295.5	13	12
CA199	479.85	10	13
CEA	3.675	12	13
CA125	16.35	11	13
Albumin (g/L)	39.1	12	12
NLR	1.949	12	13
LMR	4.068	12	12
PLR	136.744	12	12
NMR	13.068	11	12
SII	348.305	11	13

Considering the overlap and potential interference among neutrophil, lymphocyte, NLR, LMR, NMR and SII, we conducted multivariate analysis incorporating TNM stage, treatment, neutrophil, lymphocyte, CA199, CA125, and CEA. Furthermore, NLR, LMR, NMR, and SII were separately analyzed in combination with TNM stage and treatment in multivariate analyses (**Figure 2**). Multivariate analysis revealed that, in addition to TNM stage (I vs. III: HR = 3.052, 95% confidence interval (CI) = 1.900-4.905, *P* < 0.001; I vs. IV: HR = 4.815, 95% CI = 2.504–9.258, *P* < 0.001), which are closely related to the prognosis of PDAC patients, neutrophils (HR = 1.416, 95% CI = 1.037-1.932, *P* = 0.028), lymphocytes (HR = 0.625, 95% CI = 0.462-0.846, *P* = 0.002), CEA (HR = 1.820, 95% CI = 1.315-2.518, *P* < 0.001), CA125 (HR = 1.392, 95% CI = 1.001-1.936, *P* = 0.049), NLR (HR = 1.748, 95% CI = 1.210–2.525, *P* = 0.003), LMR (HR = 0.597, 95% CI = 0.430–0.829, P = 0.002), NMR (HR = 2.065, 95% CI = 1.331–3.206, P = 0.001), and SII (HR = 1.751, 95% CI = 1.244–2.466, *P* = 0.001) are also significantly associated with the prognosis of PDAC patients.

Notably, after multivariate analysis, the association between CA199 levels and prognosis in PDAC patients became less significant compared to that of CEA. The lymphocyte counts showed a negative correlation with prognosis in both univariate and multivariate analyses.

### Prognostic LASSO coefficient screening

**Figure 3:**
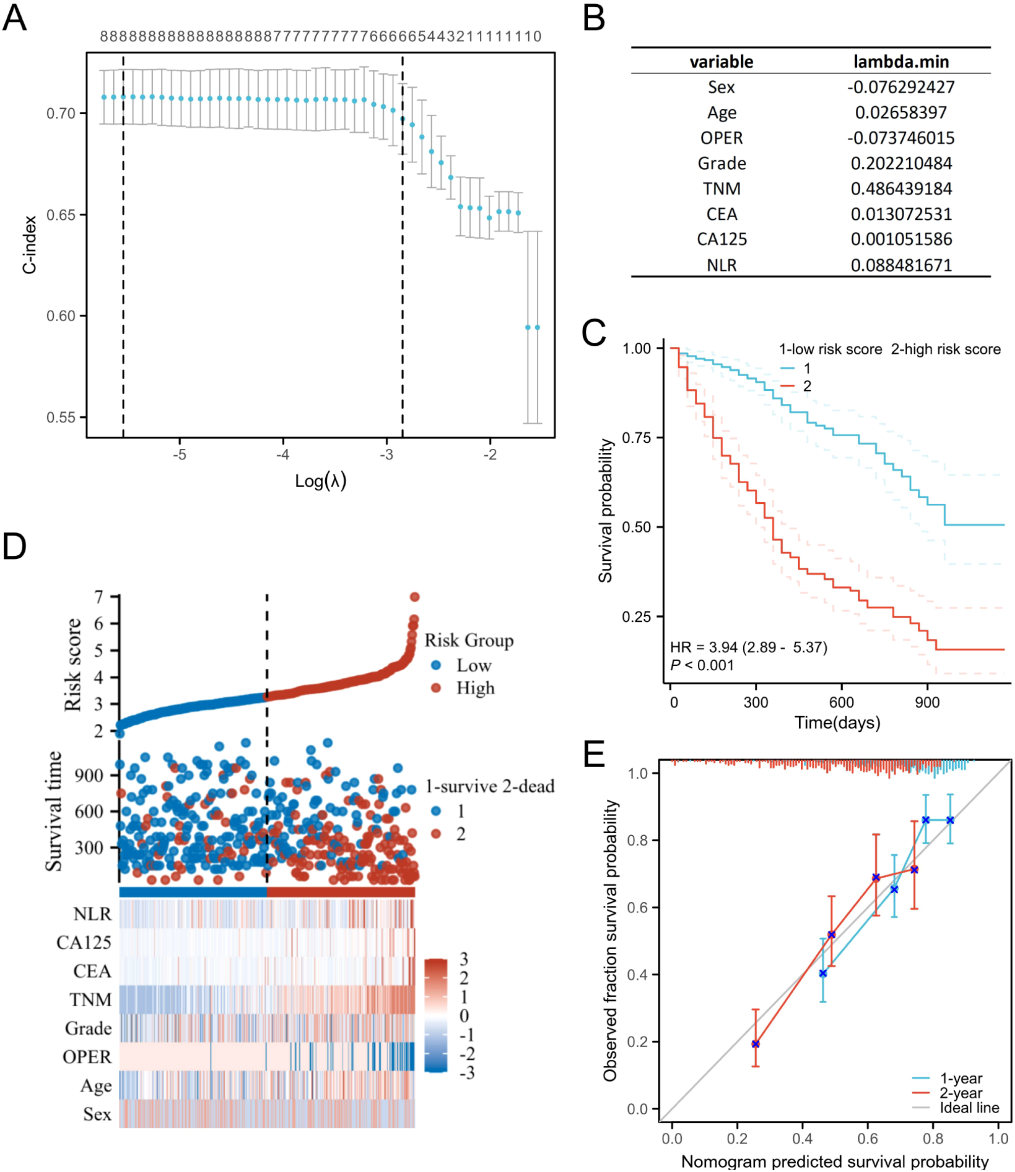
**(A, B)** Prognostic LASSO coefficient for PDAC patients. **(C)** Kaplan-Meier curve of low risk score and high risk score. **(D)** Risk factor diagram for PDAC patients based on the new risk scoring model. **(E)** The prognostic calibration curve of the new risk scoring model.

To establish a prognostic model for PDAC patients, we attempted to combine the clinical characteristics—such as gender, age, TNM stage, histologic grade, and treatment—with previously validated independent prognostic factors, including neutrophil, lymphocyte, CEA, CA125, NLR, LMR, NMR, and SII in different combinations. Given the established importance of CA199 in pancreatic cancer, it was also included and subjected to multiple combinations. By calculating the LASSO coefficients, we aimed to obtain an optimal prognostic prediction model. When the value of Log(λ) is small (the left part of the graph), the C-index is relatively high and stable, remaining 0.73, indicating that the model has good predictive accuracy (**Figure 3**). Based on this, we established a risk scoring model for prognostic prediction in PDAC patients.

Risk score = NLR*0.088 + CA125*0.001 + CEA*0.013 + TNM*0.486 + Grade*0.202 - OPER*0.074 + Age*0.026 - Sex*0.076

It is worth noting that radical surgery was represented by 1 and palliative chemotherapy by 0, with a correlation coefficient of -0.074 for prognosis, indicating that radical surgery can reduce the risk of adverse outcomes associated with prognosis. Meanwhile, male was represented by 1 and female by 2, with a correlation coefficient of -0.076 for prognosis, indicating that male patients have higher risk of poor prognosis than female.

We validated the effectiveness of this prognostic model by plotting a risk factor diagram. We stratified the risk scores into high-risk score and low-risk score groups based on the highest Youden index 3.41. Survival analysis demonstrated that the low-risk score group had a significantly better prognosis compared to the high-risk score group (HR = 3.94, 95% CI = 2.89–5.37, *P* < 0.001). The prognostic calibration curve also demonstrates that the model accurately predicts the 1-year and 2-year survival rates of PDAC patients (**Figure 3**).

## Discussion

Pancreatic cancer is known for its challenging diagnosis and high malignancy, leading to an extremely poor prognosis. The median survival time for PDAC patients is one year (15). Most patients are diagnosed at advanced stages, with only a small fraction eligible for radical pancreatectomy, which can offer a longer survival period (16). However, these patients still face significant perioperative mortality risks, and some may experience recurrence and metastasis within months after surgery (17, 18). It is particularly important to establish a simple and accessible model that can accurately predict the prognosis of PDAC patients, providing a basis for the selection of treatment strategies.

Immune cells including neutrophils, lymphocytes, are closely associated with cancer initiation, progression, and prognosis. Neutrophils can have an immunosuppressive role in cancer that promotes tumor growth, primarily by dampening the recruitment of other immune cells to the tumor microenvironment (19). Lymphocytes play a vital role in the antitumor immune response by inhibiting tumor growth, and the peripheral blood lymphocyte count can act as a marker of the body’s immune status (20). Studies have confirmed that inflammatory markers, such as neutrophil, lymphocyte, as well as immune-inflammatory scores such as, NLR, LMR, NMR and SII, are associated with the prognosis of pancreatic cancer patients. High SII, PLR, and NLR primarily indicate increased neutrophils and platelets along with decreased lymphocytes, which means the patient has a weakened immune response and an enhanced inflammatory response, leading to a poorer prognosis (21). This is consistent with our study.

In our study, univariate analysis demonstrated a correlation between CA199 and the OS (HR: 2.199 (1.604 - 3.015), p < 0.001) of PDAC patients. However, in the multivariate analysis incorporating factors such as TNM stage and so on, the association of CA199 with OS (HR: 1.335 (0.943 - 1.890), p = 0.104) was weaker compared to that of CEA (HR: 1.820 (1.315 - 2.518), P < 0.001). CA19–9 is a modified Lewis(a) blood group antigen, which is produced by exocrine epithelial cells. The antigen is the only biomarker approved by the FDA for the diagnosis, treatment evaluation, and postoperative recurrence monitoring of PDAC. However, 5–10% of PDAC patients are unable to synthesize CA19–9 due to their Lewis antigen-negative blood type. Additionally, CA19–9 is expressed in normal epithelial cells of the pancreas, bile duct, stomach, and colon, which may lead to false-positive results. One of the common symptoms of PDAC is obstructive jaundice caused by cholestasis. In such cases, elevated serum CA19–9 levels may result from biliary obstruction, malignancy, or a combination of both. Therefore, the accuracy of CA19–9 varies with disease stage. In the multivariate analysis, we have already incorporated the TNM staging factor, which may weaken the correlation between CA19–9 and prognosis (22, 23). Studies indicate that CA19–9 has a sensitivity of 70%, specificity of 87%, and accuracy of 84%, yet its positivity rate is only 65% in patients with resectable PDAC. Additionally, CA19–9 levels are significantly elevated in advanced stages of PDAC, while in prospective studies of asymptomatic populations, its positive predictive value is merely 0.5–0.9% (24, 25). This result was also validated when calculating the LASSO coefficients for prognosis. When we incorporated CEA, CA125, CA199, NLR, and clinical characteristics — such as gender, age, TNM stage, histologic grade, and treatment, to calculate the LASSO coefficients, CA199 demonstrated an extremely minor influence on prognosis (0.00003033).

CEACAM5 (CEA) is a 180–200 kDa glycoprotein derived from endodermally derived epithelial tissues, primarily found in the gastrointestinal, urinary, and respiratory tracts. Serum CEA levels are elevated in 30–60% of PDAC patients. Although CEA has lower sensitivity and specificity as a standalone prognostic marker compared to CA19-9, a multi−biomarker panel demonstrates superior prognostic performance over single biomarkers in PDAC (26). We incorporated CEA, CA125, NLR, and clinical characteristics to establish a new prognostic prediction model. Meanwhile, we validated the high efficacy of this model. To our knowledge, this is the first attempt to develop a PDAC prognostic model that combines inflammatory markers and tumor markers. However, some limitations also remains in this study. Firstly, this study is retrospective, which may introduce selection bias, and the sample size is limited. Future prospective studies with larger cohorts should be conducted to validate the findings. Secondly, our data were collected from a single center. We could not perform external validation. To strengthen the credibility of our new model, it is essential to collect data from multiple centers and conduct external validation.

## Conclusions

In this retrospective study, we explored the relationship between pre-treatment blood markers and clinical characteristics with OS in PDAC patients. Multivariate modeling also reveals that, when combined with other factors, CEA may have greater prognostic value than CA199. We developed a new prognostic model based on blood markers and clinical characteristics, which is simple and accessible. This enables us to conduct more precise prognostic assessments and to make informed treatment decisions.

## Data Availability

The raw data supporting the conclusions of this article will be made available by the authors, without undue reservation.
